# 多发肺结节的基础研究和临床治疗进展

**DOI:** 10.3779/j.issn.1009-3419.2019.03.10

**Published:** 2019-03-20

**Authors:** 雪杰 吴, 东来 陈, 蓉英 朱, 一飞 王, 昶 陈, 勇兵 陈, 文涛 杨

**Affiliations:** 1 215000 苏州，苏州大学附属第二医院胸心外科 Department of Thoracic Surgery, the Second Affiliated Hospital of Soochow University, Suzhou 215000, China; 2 200433 上海，同济大学附属上海市肺科医院胸外科 Department of Thoracic Surgery, Shanghai Pulmonary Hospital, Tongji University School of Medicine, Shanghai 200433, China

**Keywords:** 肺肿瘤, 多发肺结节, 基因组学, 外科治疗, Lung neoplasms, Multiple pulmonary nodules, Genomic sciences, Surgical treatment

## Abstract

肺癌是世界上发病率和死亡率最高的肿瘤。随着多层螺旋计算机断层扫描（computed tomography, CT）技术的发展和肺癌筛查的广泛开展，越来越多的肺结节被发现, 其中不少是多发肺结节，这些结节在病理学上常被诊断为多原发肺腺癌。对于具有不同影像学特征的多发结节，首选处理方法不尽相同，且每个肺结节的处理方法仍存在很大争议。近年来多发肺结节各病灶的演进及病灶间的相互影响机制，病灶内和病灶间肿瘤细胞在基因组学方面的同质性和异质性也备受关注。本文从组织病理学、基因组学、外科处理等多方面综合论述多发肺结节的研究进展。

## Abstract (en)

Lung cancer leads to the highest cancer-related morbidity and mortality worldwide. With the development of multi-slice spiral computed tomography technology and the implement of lung cancer screening, more and more lung nodules have been discovered, many of which are multiple pulmonary nodules. These pulmonary nodules are usually diagnosed as multiple primary lung adenocarcinomas from a pathological perspective. For multiple nodules with different imaging features, the preferred treatment methods are different, and the treatment of each lung nodule is still controversial. In recent years, the interactions between multiple lesions and the evolution of the lesions as well as the inter-tumoral and intratumoral homogeneity and heterogeneity of the genomics also arouse attention. Our review gathered the research progress in multiple pulmonary nodules from the points of histopathology, genomics and surgical management.

随着人们的健康意识的提高以及薄层计算机断层扫描（computed tomography, CT）技术的发展，肺癌中表现为磨玻璃结节的病例越来越多。近年，在不吸烟和女性人群中，肺多发结节的检出率大大增加。多发肺结节的自然史和单发病变的区别还没有得到充分的研究，也没有明确如何管理这些患者。现有的处理策略很多基于以前的报告，其中只包含少量的多发肺结节病例。本文旨在归纳多发肺结节的自然史和基因组特征，整理汇总可能改善这些患者预后及诊疗策略的文献进展。

## 1 多发肺结节的定义及影像学表现

多发肺结节是指肺内存在两个或以上直径均≤3 cm的类圆形或不规则形病灶。其影像学多表现为若干个含磨玻璃影的结节。磨玻璃影（ground-glass opacity, GGO）是指肺内模糊的、密度轻度增高的云雾状淡薄影/圆形影，其内还可见支气管征象和血管征象^[1]^。根据结节内是否含有实性成分，将其分为纯磨玻璃结节（pure ground-grass nodules, pGGNs），部分实性结节（part-solid nodules）和纯实性结节（solid nodule），纯实性结节表现为结节内均一的高密度软组织影，其内血管及支气管影像被掩盖。部分实性结节是指既包含磨玻璃影又包含实性软组织影的结节，其中部分实性结节的恶性概率最高，依次为磨玻璃结节及实性结节^[2]^。多发肺结节常提示早期多原发肺癌（multiple primary lung cancer），且病理上常提示早期肺腺癌。在多发结节中直径最大的病灶称为主病灶（primary lesions），其余的称为次病灶（secondary lesions）。

## 2 组织病理学演进

第八版AJCC分期手册（American Joint Committee On Cancer Staging Manual）将多原发肺癌分为四种疾病模式，第一种为两个或两个以上的组织学类型不同的肿块；第二种为多个磨玻璃或部分实性结节，组织学上均呈鳞屑样生长模式；第三种为磨玻璃影和实性影构成的斑片状区域，组织学上多为粘液性浸润性腺癌；第四种为肺内转移瘤^[3]^。多发肺结节的主要病理类型为肺腺癌相关的组织亚型^[4]^。其涉及的组织亚型涵盖了从不典型腺瘤样增生（atypical adenomatous hyperplasia, AAH）到原位腺癌（adenocarcinoma *in situ*, AIS）到微浸润腺癌（microinvasive lung adenocarcinoma, MIA）再到浸润性腺癌（invasive adenocarcinoma, IA）等多个腺癌演进的阶段^[5, 6]^。

## 3 基因组学特征

在AAH病变中，最常发生突变的基因并不是经典的驱动基因，而是DNA修复和染色质重塑的相关基因^[7]^，这表明这些病变倾向于克隆扩增和继发性基因突变。在AIS中，包括更多驱动突变，如表皮生长因子受体（epidermal growth factor receptor, *EGFR*）突变和KRAS。在MIA中，甚至存在更多的驱动基因，但主要在肿瘤的密集或侵入区域。杂合性缺失（loss of heterozygosity, LOH）是肿瘤中遗传改变的间接指标^[8]^。研究^[9]^表明3p和9p染色体的LOH可能是癌症发生的早期事件。Takamochi等^[10]^分析了11例患者体内的18个AAH和19个腺癌病灶，表明LOH在染色体9q和16p之间存在因果关系。虽然这些基因组改变存在于许多前驱病变，使用LOH分析来确定肿瘤是否为单一细胞起源仍具挑战性。在对术后病理类型为腺癌的78例患者的159个病灶分析发现其中38例患者存在*EGFR*突变，在38对配对病变中，92.1%的配对病变呈现不同的*EGFR*突变状态，同时，各个肿瘤的体积和直径与L858R突变相关^[4, 11-13]^。因此，多原发肺腺癌很可能存在着不同的细胞起源。在对15例多发同步肺腺癌的基因组分析中发现^[14]^，虽然有共同的遗传背景和接触史，但所有的肿瘤有不同的基因组谱，包括体细胞点突变、拷贝数畸变、染色体结构变异甚至突变。这些数据提供了在独立的肿瘤发展过程中多个突变过程发挥作用的证据。统筹基因组学和组织病理学评估对于区分多原发性肿瘤和大多数患者的肺内转移是极具价值的。此外，多原发肿瘤间往往具有不同的基因组特征，而转移瘤通常保留了原发性肿瘤大量的基因组畸变^[15-17]^。因此，在外显子层面进行全面的基因组分析，可以为临床和组织学评估提供关键信息，从而准确区分多个原发性肺癌和肺内转移。当然，一个重要的问题是肿瘤内部的异质性。据报道，非小细胞肺癌（non-small cell lung cancer, NSCLC）的*EGFR*突变在6.3%-30%的病例中显示出肿瘤内部的异质性^[18, 19]^。另一方面，尽管细胞在肿瘤发生过程中所采取的突变路线是随机的，但遗传特点可能受到某个关键信号通路功能选择的限制，随着时间的推移，动态突变过程会形成亚克隆基因组，这可能会导致在多原发肺癌中观察到的大量内部异质性^[20]^。同时，他们证实这些多原发肿瘤是通过完全不同的遗传事件的积累而发展形成的，没有共同的结构改变。此外，他们又进行了一系列实验，说明了受体酪氨酸激酶在肺癌演进中所起的中心作用，并认为多原发肺癌中独立肿瘤的遗传特征可能会由于关键的致癌途径的优先激活而受到限制。我们从有限的文献回顾中不难得出，分子检测不仅可以提供鉴别诊断和治疗方案，而且还可能提供预后价值，因为具有相同突变的多灶病变有可能随着时间的推移获得类似转移性疾病的表型。

## 4 外科处理

多原发肺癌的最高T分期和淋巴结情况是预后的主要因素^[21]^，因此对于多发肺结节，应优先切除主病灶，其次根据情况考虑次病灶。虽然手术治疗多个病灶常取决于它们的解剖位置、大小和数量，以及患者的年龄和肺功能；但是，同时切除多个GGNs仍然存在争议，目前仍没有已经明确的标准来选择手术方式，也没有残留结节的后续处理原则；结节大小是多原发/同时性pGGNs恶变的独立危险因素，其阳性界限值为9.4 mm，此时积极的早期外科干预是有必要的^[22]^。在多个GGNs中，实占比（consolidation/tumor ratio, CTR）与淋巴结转移密切相关，主病灶CTR > 0.5为无复发生存期（relapse free survival, RFS）的独立危险因子^[23]^。Shimada等^[24]^通过分析大量病例提出了他们关于同侧多发肺结节的基本策略：当肿瘤在同侧肺部时，应全部切除病灶；10 mm以上或影像学上实性成分为主的病灶，应通过单边手术治疗。对于次病灶，10 mm以下或影像学上呈磨玻璃样的病灶，通常病灶生长缓慢，以手术切除为主。如果预期切除范围扩大，应有限切除恶性肿瘤及容易处理的小结节。如果有多个病灶散落在多个肺叶中，应手术切除直径为10 mm以上或新出现的实性小结节。根据经验，如果多发GGNs在同一肺叶，可行楔形切除、肺段切除术或肺叶切除术；若分布在同侧多叶肺中。手术入路应根据病变部位、多楔形切除或肺段切除，尽可能保留肺功能；如果多个GGNs在两侧肺，则可行同期或双期胸腔镜手术；但一般情况考虑双期手术，除非病人能够耐受同期手术切除^[25, 26]^。即使同期切除，肺的总切除范围也不要超过10个肺段^[26]^。另外，多发GGNs手术切除后的预后多令人满意，且亚肺叶切除也不影响多发GGNs的预后。患者的总体生存率在主病灶切除后并不受残余亚结节的增长和新结节出现的影响^[27]^。Ye等^[28]^报告了1例罕见的多原发肺肿瘤，患者具有结节间的异质性，其中患者接受了吉非替尼不敏感的外周病灶切除，再继续使用吉非替尼持续治疗敏感的双侧多发性GGNs，病人获得了完全缓解并得到了1.5年的无病生存期，这种新颖的外科治疗策略可能是一种有希望治疗同步多原发肺癌的方法。

## 5 总结与展望

目前对于多发肺结节，并没有完整且具有指导性的临床诊治指南，对于亚厘米pGGNs以及主灶切除后余下结节的处理仍具有争议；对于那些年龄大且肺功能不良的患者，立体定向放射治疗（stereotactic body radiation therapy, SBRT）用于治疗无法手术的多原发性肺癌，似乎是一种可行的方法。早期肺癌手术切除和SBRT在总体生存率（overall survival, OS）、癌症特异性生存期（cancer-specific survival, CSS）、无病生存率（disease-free survival, DFS）、局部控制（local control, LC）和远处控制（distant control, DC）之间的差异，发现1年、3年的各项数据基本一致，表明SBRT与早期肺癌手术切除具有相似的预后效果^[29]^。对于接受胸部放射治疗的多原发肺癌患者，SBRT也取得了良好的效果^[30]^。Sinha等^[31]^分析了8例接受SBRT治疗的多原发肺癌患者，结果显示均没有疾病的进展。随着更深入的研究开展，SBRT在多发肺结节的临床处理中会更具有积极意义。

对于多发肺结节患者首次发现及在后续的随访过程中，无论是穿刺活检还是手术活检都非无创，如何通过无创的方式来实现对多发肺结节综合评估是未来肿瘤研究发展的趋势。被称为“液体活检”的外周血循环肿瘤DNA（circulating tumor DNA, ctDNA）和外泌体检测，极大地推动了肿瘤无创检测的进展。一种更具特异性的ctDNA检测方法——CAPP-Seq^[32]^，可以在100%的Ⅱ期NSCLC患者和50%的Ⅰ期患者中检测到ctDNA，同时CAPP-Seq可以评估无活组织肿瘤的筛选和基因分型；通过比较112例NSCLC患者、60例肺炎患者和40例体检健康者的外泌体miRNA，发现NSCLC组血清外泌体miR-184表达量明显高于肺炎组和体检健康者（*P* < 0.05），miRNA对NSCLC的诊断敏感性为92.54%，特异性为84.19%^[33]^；Mei^[34]^通过分析32例肺癌患者外周血有核细胞的染色体3pLOH发现，外周血有核细胞的3pLOH联合检出率为62.625%，而空白对照组均未检出LOH。这些与基因组有关的检测对于无创鉴别多发肺结节的良恶性又给出了一种新思路。在当今“精准医疗”理念如火如荼推广的背景下，基因组学尤其是遗传学相关特征与临床治疗的关系日趋紧密。如何通过基因组等技术结合多学科，为各个患者制定更无创的综合评估以及个性化治疗方案，提高疾病诊治与预防的效益，是值得广大临床医生思考的问题。未来，ctDNA、外泌体和LOH的检测等更多的无创检查手段可以常规应用于临床检测和监测，成为有实用价值的临床肿瘤诊断项目。可以预期，随着多发结节研究的广泛展开和不断深入，其处理原则也必定会向着更精准、更无创及个体化的方向发展。
